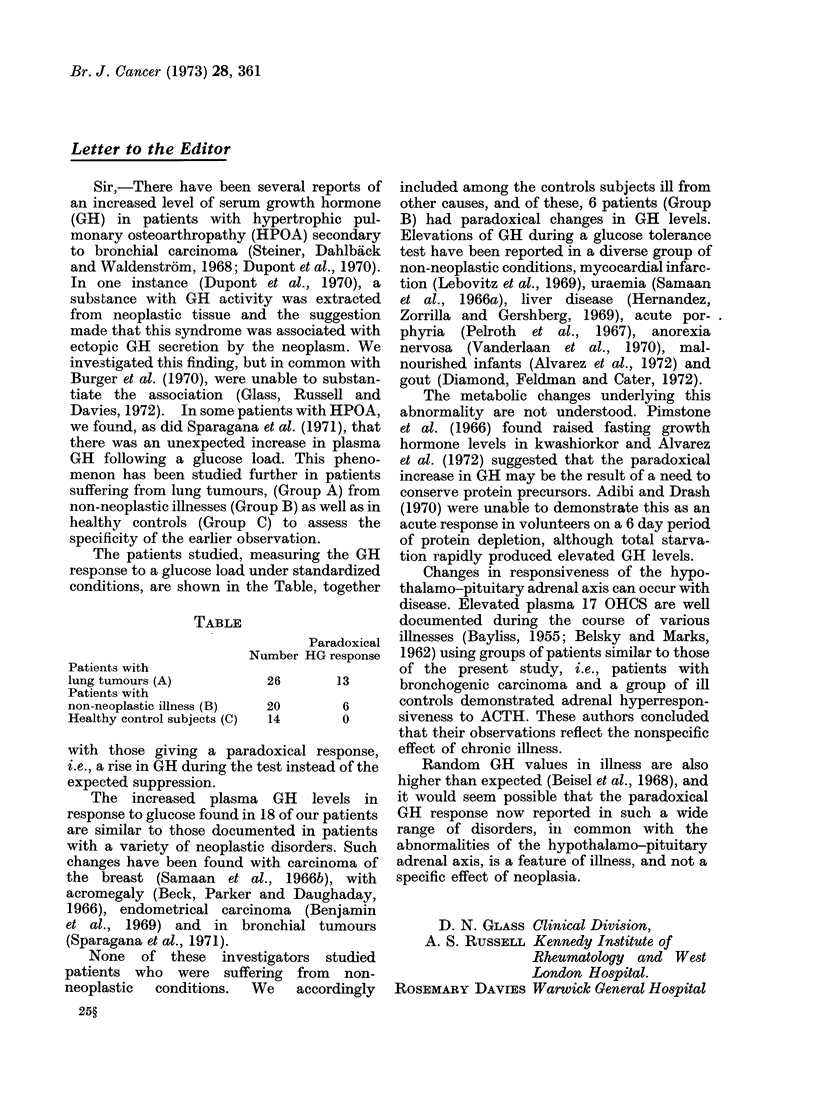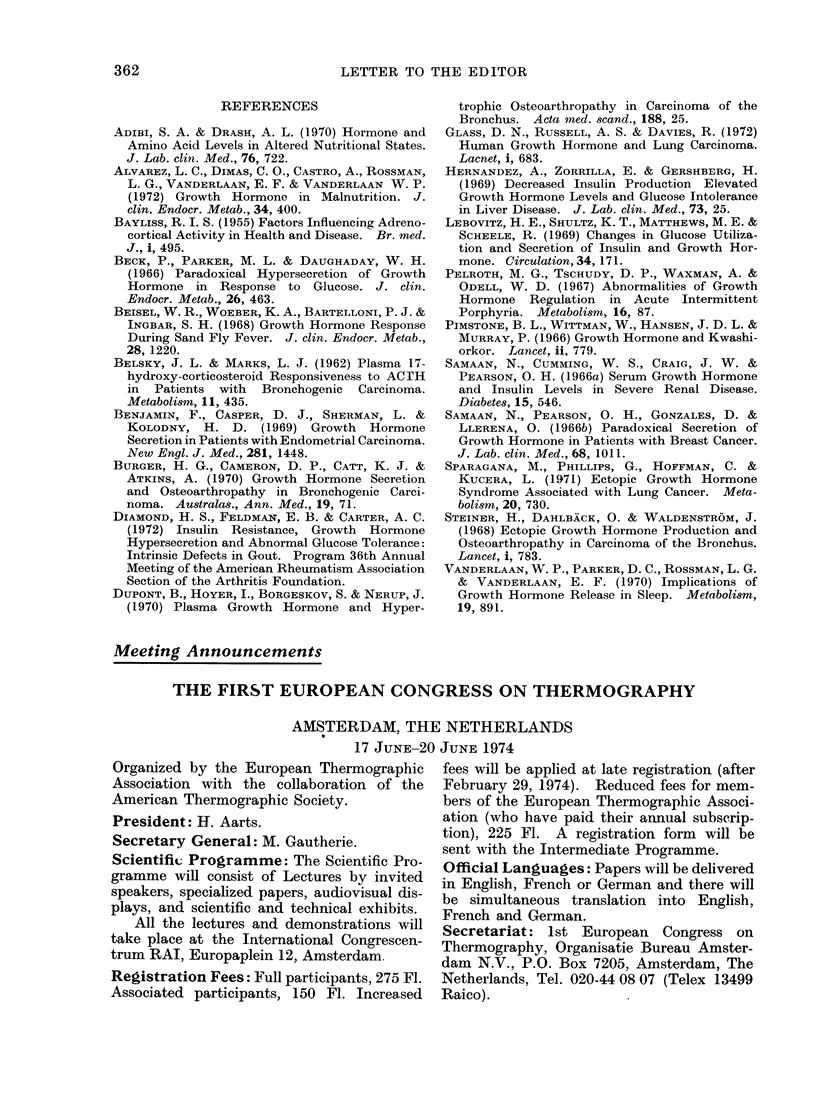# Letter: Growth hormone response in disease.

**DOI:** 10.1038/bjc.1973.161

**Published:** 1973-10

**Authors:** D. N. Glass, A. S. Russell, R. Davies


*Br. J. Cancer* (1973) **28**, 361

### *Letter to the Editor*

Sir,—There have been several reports of an increased level of serum growth hormone (GH) in patients with hypertrophic pulmonary osteoarthropathy (HPOA) secondary to bronchial carcinoma (Steiner, Dahlbäck and Waldenström, 1968; Dupont *et al.*, 1970). In one instance (Dupont *et al.*, 1970), a substance with GH activity was extracted from neoplastic tissue and the suggestion made that this syndrome was associated with ectopic GH secretion by the neoplasm. We investigated this finding, but in common with Burger *et al.* (1970), were unable to substantiate the association (Glass, Russell and Davies, 1972). In some patients with HPOA, we found, as did Sparagana *et al.* (1971), that there was an unexpected increase in plasma GH following a glucose load. This phenomenon has been studied further in patients suffering from lung tumours, (Group A) from non-neoplastic illnesses (Group B) as well as in healthy controls (Group C) to assess the specificity of the earlier observation.

The patients studied, measuring the GH response to a glucose load under standardized conditions, are shown in the Table, together with those giving a paradoxical response, *i.e.*, a rise in GH during the test instead of the expected suppression.

TABLE

| | Number | Paradoxical HG response |
|---|---|---|
| Patients with lung tumours (A) | 26 | 13 |
| Patients with non-neoplastic illness (B) | 20 | 6 |
| Healthy control subjects (C) | 14 | 0 |

The increased plasma GH levels in response to glucose found in 18 of our patients are similar to those documented in patients with a variety of neoplastic disorders. Such changes have been found with carcinoma of the breast (Samaan *et al.*, 1966*b*), with acromegaly (Beck, Parker and Daughaday, 1966), endometrical carcinoma (Benjamin *et al.*, 1969) and in bronchial tumours (Sparagana *et al.*, 1971).

None of these investigators studied patients who were suffering from non-neoplastic conditions. We accordingly included among the controls subjects ill from other causes, and of these, 6 patients (Group B) had paradoxical changes in GH levels. Elevations of GH during a glucose tolerance test have been reported in a diverse group of non-neoplastic conditions, mycocardial infarction (Lebovitz *et al.*, 1969), uraemia (Samaan *et al.*, 1966*a*), liver disease (Hernandez, Zorrilla and Gershberg, 1969), acute porphyria (Pelroth *et al.*, 1967), anorexia nervosa (Vanderlaan *et al.*, 1970), malnourished infants (Alvarez *et al.*, 1972) and gout (Diamond, Feldman and Cater, 1972).

The metabolic changes underlying this abnormality are not understood. Pimstone *et al.* (1966) found raised fasting growth hormone levels in kwashiorkor and Alvarez *et al.* (1972) suggested that the paradoxical increase in GH may be the result of a need to conserve protein precursors. Adibi and Drash (1970) were unable to demonstrate this as an acute response in volunteers on a 6 day period of protein depletion, although total starvation rapidly produced elevated GH levels.

Changes in responsiveness of the hypothalamo–pituitary adrenal axis can occur with disease. Elevated plasma 17 OHCS are well documented during the course of various illnesses (Bayliss, 1955; Belsky and Marks, 1962) using groups of patients similar to those of the present study, *i.e.*, patients with bronchogenic carcinoma and a group of ill controls demonstrated adrenal hyperresponsiveness to ACTH. These authors concluded that their observations reflect the nonspecific effect of chronic illness.

Random GH values in illness are also higher than expected (Beisel *et al.*, 1968), and it would seem possible that the paradoxical GH response now reported in such a wide range of disorders, in common with the abnormalities of the hypothalamo–pituitary adrenal axis, is a feature of illness, and not a specific effect of neoplasia.

D. N. GLASS *Clinical Division,*
A. S. RUSSELL *Kennedy Institute of Rheumatology and West London Hospital.*
ROSEMARY DAVIES *Warwick General Hospital*

REFERENCES

ADIBI, S. A. & DRASH, A. L. (1970) Hormone and Amino Acid Levels in Altered Nutritional States. *J. Lab. clin. Med.*, **76**, 722.

ALVAREZ, L. C., DIMAS, C. O., CASTRO, A., ROSSMAN, L. G., VANDERLAAN, E. F. & VANDERLAAN W. P. (1972) Growth Hormone in Malnutrition. *J. clin. Endocr. Metab.*, **34**, 400.

BAYLISS, R. I. S. (1955) Factors Influencing Adrenocortical Activity in Health and Disease. *Br. med. J.*, **i**, 495.

BECK, P., PARKER, M. L. & DAUGHADAY, W. H. (1966) Paradoxical Hypersecretion of Growth Hormone in Response to Glucose. *J. clin. Endocr. Metab.*, **26**, 463.

BEISEL, W. R., WOEBER, K. A., BARTELLONI, P. J. & INGBAR, S. H. (1968) Growth Hormone Response During Sand Fly Fever. *J. clin. Endocr. Metab.*, **28**, 1220.

BELSKY, J. L. & MARKS, L. J. (1962) Plasma 17-hydroxy-corticosteroid Responsiveness to ACTH in Patients with Bronchogenic Carcinoma. *Metabolism*, **11**, 435.

BENJAMIN, F., CASPER, D. J., SHERMAN, L. & KOLODNY, H. D. (1969) Growth Hormone Secretion in Patients with Endometrial Carcinoma. *New Engl. J. Med.*, **281**, 1448.

BURGER, H. G., CAMERON, D. P., CATT, K. J. & ATKINS, A. (1970) Growth Hormone Secretion and Osteoarthropathy in Bronchogenic Carcinoma. *Australas., Ann. Med.*, **19**, 71.

DIAMOND, H. S., FELDMAN, E. B. & CARTER, A. C. (1972) Insulin Resistance, Growth Hormone Hypersecretion and Abnormal Glucose Tolerance: Intrinsic Defects in Gout. Program 36th Annual Meeting of the American Rheumatism Association Section of the Arthritis Foundation.

DUPONT, B., HOYER, I., BORGESKOV, S. & NERUP, J. (1970) Plasma Growth Hormone and Hypertrophic Osteoarthropathy in Carcinoma of the Bronchus. *Acta med. scand.*, **188**, 25.

GLASS, D. N., RUSSELL, A. S. & DAVIES, R. (1972) Human Growth Hormone and Lung Carcinoma. *Lacnet*, **i**, 683.

HERNANDEZ, A., ZORRILLA, E. & GERSHBERG, H. (1969) Decreased Insulin Production Elevated Growth Hormone Levels and Glucose Intolerance in Liver Disease. *J. Lab. clin. Med.*, **73**, 25.

LEBOVITZ, H. E., SHULTZ, K. T., MATTHEWS, M. E. & SCHEELE, R. (1969) Changes in Glucose Utilization and Secretion of Insulin and Growth Hormone. *Circulation*, **34**, 171.

PELROTH, M. G., TSCHUDY, D. P., WAXMAN, A. & ODELL, W. D. (1967) Abnormalities of Growth Hormone Regulation in Acute Intermittent Porphyria. *Metabolism*, **16**, 87.

PIMSTONE, B. L., WITTMAN, W., HANSEN, J. D. L. & MURRAY, P. (1966) Growth Hormone and Kwashiorkor. *Lancet*, **ii**, 779.

SAMAAN, N., CUMMING, W. S., CRAIG, J. W. & PEARSON, O. H. (1966*a*) Serum Growth Hormone and Insulin Levels in Severe Renal Disease. *Diabetes*, **15**, 546.

SAMAAN, N., PEARSON, O. H., GONZALES, D. & LLERENA, O. (1966*b*) Paradoxical Secretion of Growth Hormone in Patients with Breast Cancer. *J. Lab. clin. Med.*, **68**, 1011.

SPARAGANA, M., PHILLIPS, G., HOFFMAN, C. & KUCERA, L. (1971) Ectopic Growth Hormone Syndrome Associated with Lung Cancer. *Metabolism*, **20**, 730.

STEINER, H., DAHLBÄCK, O. & WALDENSTRÖM, J. (1968) Ectopic Growth Hormone Production and Osteoarthropathy in Carcinoma of the Bronchus. *Lancet*, **i**, 783.

VANDERLAAN, W. P., PARKER, D. C., ROSSMAN, L. G. & VANDERLAAN, E. F. (1970) Implications of Growth Hormone Release in Sleep. *Metabolism*, **19**, 891.